# Biomarker discovery for non-invasive diagnosis of inflammatory bowel disease using blood transcriptomics

**DOI:** 10.3389/fimmu.2025.1570374

**Published:** 2025-06-20

**Authors:** Mohammad Hossein Derakhshan Nazari, Mahsa Ghorbaninejad, Shabnam Shahrokh, Anna Meyfour

**Affiliations:** ^1^ Basic and Molecular Epidemiology of Gastrointestinal Disorders Research Center, Research Institute for Gastroenterology and Liver Diseases, Shahid Beheshti University of Medical Sciences, Tehran, Iran; ^2^ Gastroenterology and Liver Diseases Research Center, Research Institute for Gastroenterology and Liver Diseases, Shahid Beheshti University of Medical Sciences, Tehran, Iran

**Keywords:** inflammatory bowel disease (IBD), diagnosis, transcriptomics, biomarker, whole blood, machine learning, bioinformatics

## Abstract

**Introduction:**

Colonoscopy remains the gold standard for diagnosing inflammatory bowel disease (IBD) even though it is an invasive and costly procedure. To enable non-invasive diagnosis, we aimed to identify blood-based transcriptomic biomarkers that specifically distinguish IBD from healthy and inflammatory controls.

**Methods:**

Public microarray and RNA-seq datasets from whole blood of IBD, rheumatoid arthritis (RA), and control subjects were analyzed. RA was included as a positive control for systemic inflammation to filter out non-IBD-specific gene signatures. Differentially expressed genes (DEGs) were identified, followed by immune cell deconvolution (CIBERSORTx), pathway and network analysis, and diagnostic model construction using LASSO and SVM. A real-life cohort (36 IBD patients, 30 controls) was recruited for qRT-PCR validation.

**Results:**

IBD blood transcriptomes exhibited altered immune profiles, including increased M0 macrophages, Tregs, and CD4 naïve T cells, and decreased memory B and activated NK cells. After excluding RA-overlapping DEGs, 25 IBD-specific DEGs with |log2FC| > 0.5 were prioritized. LASSO and SVM identified a three-mRNA panel—IL4R, EIF5A, and SLC9A8—which achieved 84% diagnostic accuracy in the discovery cohort and 99% accuracy in the real-life cohort. Network analysis highlighted NDUFB2 as a key downregulated hub gene linked to mitochondrial complex I dysfunction and oxidative phosphorylation disruption. Elevated oxidative stress in IBD was confirmed by increased Total Oxidant Status (TOS) levels in patient plasma.

**Discussion:**

Our findings support the use of peripheral blood transcriptomics for IBD diagnosis and demonstrate that a focused three-gene panel can achieve high diagnostic accuracy. The inclusion of RA as an inflammatory control enabled the identification of IBD-specific markers, minimizing confounding from general immune activation. These results provide a practical foundation for developing non-invasive diagnostic tools for clinical use.

## Introduction

Inflammatory bowel disease (IBD), including Crohn’s disease (CD) and ulcerative colitis (UC), is a chronic, debilitating, incurable, and immune-mediated disorder that commonly emerge with abdominal pain, diarrhea, and blood in the stool (1). The primary diagnostic indicator for IBD is intestinal inflammation (2). IBD is differentiated from other gastrointestinal disorders primarily based on clinical symptoms and the use of diagnostic imaging like CT scans and sonography, along with routine lab tests (3). However, the accurate diagnosis of IBD in patients who have been excluded from having other conditions still relies on colonoscopy—an invasive procedure where gastroenterologists insert a scope into the colon of patients (occasionally anesthetized) through the anus to capture images and obtain biopsies (4, 5). This method can exacerbate inflammation and carries several potential side effects, such as bleeding, and still there is a need for an accurate, reliable, and non-invasive test that can make the detection of IBD more feasible. Liquid biopsy is a non-invasive diagnostic method that analyzes various markers present in bodily fluids such as blood, urine, cerebrospinal fluid, and saliva. In contrast to solid biopsies, liquid biopsies are more accessible source of cells that could be used to differentiate between disease and controls (6).

In this study, transcriptome profiles of peripheral blood samples from IBD patients were analyzed using a combination of machine learning algorithms, bioinformatics tools, and experimental approaches to identify discriminative biomarkers for an IBD-specific diagnostic panel. To enhance specificity, gene expression profiles of rheumatoid arthritis (RA)—commonly used as a positive control for systemic inflammation in biomedical research due to shared Th1/Th17 response pathways cytokine profiles (IL-17, TNF-α, and IFN-γ) with IBD (7–9)—were also examined. Also, to further understand the IBD pathogenesis in blood, functional analysis including immune cell landscape analysis, network analysis, and pathway annotation was performed.

## Materials and methods

### Collecting cohorts

Using Gene Expression Omnibus (GEO), we searched for datasets containing whole blood samples from patients with IBD, RA, and healthy individuals. For the discovery cohort, we identified GSE94648 (10) and GSE119600 (11), which include samples from healthy individuals and patients with IBD. GSE93272 (12) was selected for comparing RA patients and control individuals. For validation, GSE169568 (13) and GSE166924 (14), both containing whole blood samples of IBD patients and non-IBD controls (including healthy individuals) were chosen as test cohorts to evaluate the marker genes identified in the discovery phase. We only included IBD patients with confirmed active disease and no prior exposure to antibody treatments. Series matrices and metadata for GSE94648, GSE119600, GSE93272, and GSE169568, as well as the raw count matrix for GSE166924, were downloaded from GEO. Probe IDs were converted to gene symbols using annotation files obtained from GEO.

### Integrating IBD discovery cohorts by meta-analysis

To create the discovery cohort, the series matrices of GSE94648 and GSE119600 were integrated. For each gene, expression levels were calculated as the mean of its transcript expressions. Using principal component analysis (PCA), outliers were identified and removed. Then, the batch effect was corrected using the *ComBat* function from the *sva* package in R. All the downstream analyses, including functional annotation, network construction, pathway analysis, and biomarker panel development were performed using discovery cohorts.

### Gene expression and annotation analysis

For the microarray datasets (GSE94648, GSE119600, GSE93272, and GSE169568), differential gene expression between cases and controls (IBD vs. control and RA vs. control) was performed using the R package *Limma*. For RNA sequencing data (GSE166924), differential gene expression analysis (IBD vs. control) was conducted using the *DESeq2* package (15) in R. Genes with a discovery rate (FDR) < 0.05 were considered as differentially expressed genes (DEG). Using the discovery cohort, Gene Ontology (GO) and pathways analyses were carried out using Gene Ontology Resource (GOR) (https://geneontology.org/) (16) and Molecular Signature (MSigDB) databases (https://www.gsea-msigdb.org/gsea/msigdb/) (17), respectively. Significant biological processes (BP), molecular functions (MF), cellular components (CC), and signaling pathways were detected using FDR < 0.05 as the criteria.

### Immune cell profile analysis

To identify changes in immune cell subpopulations in IBD patients, CIBERSORTx (http://cibersortx.stanford.edu) (18) was used. Series matrices of discovery cohorts (GSE94648 and GSE119600 for IBD and GSE93272 for RA) were converted into mixture files, which then were analyzed using the core LM22 signature containing 547 genes that ultimately discriminate 22 hematopoietic cell phenotypes. The results were reported as relative fractions for all immune cell subtypes. Statistical analysis was performed using SPSS software. Firstly, Levene’s test was conducted to ensure equal variances (19). Then, an unpaired two-tailed t-test was performed to identify significantly altered immune cell proportions.

### Network construction and annotation

Network analysis was performed to delineate the hub genes likely to play a role in IBD pathogenesis in blood. To find a network of interacting genes and their encoded proteins, we firstly measured the Pearson correlation. Using correlation |coefficients| ≥ 0.8 significant co-expression interactions were considered as edges, and the network was built in Cytoscape software (version 3.9.1) (20). Based on the degree, which is the number edges, we selected the top 50 node using the *CytoHubba* plugin (21). Then, using STRING database (https://string-db.org/) (22), protein-protein interactions (PPI) that are experimentally verified were added to the network in Cytoscape. The final network of 50 nodes, comprising co-expressions and PPIs, was analyzed by *CentiScape2.2* plugin (23) to identify regulatory nodes through centrality factors. Also, the network was analyzed using MSigDB and Pathview database (https://pathview.uncc.edu/) (24) to identify and visualize the signaling pathways associated with hub genes. In Pathview database, analysis was performed using a gene data file in which hub genes are arranged in rows, and Log_2_FC are arranged in a column.

### Biomarker identification and diagnostic panel development

To identify DEGs specific to blood inflammation in IBD, RA was used as a positive control. DEGs identified in the RA vs. control comparison were removed from the list of DEGs in IBD. From the remaining genes, those with |Log_2_FC| > 0.5 were selected to enrich genes with higher discriminative potential. Then, the Least Absolute Shrinkage and Selection Operator (LASSO) algorithm was used to identify gene biomarkers capable of distinguishing IBD from controls. The tuning parameter (λ) was estimated via 10-fold cross-validation using the cv.*glmnet* function from the *glmnet* package in R. The *COM* function from the *gtools* package was then applied to design panels of three to eight genes. To identify optimal gene sets for diagnosing IBD, a support vector machine (SVM) classifier was implemented using the *e1071* package in R. Data were split into 80% training and 20% testing sets. The classifier was trained on the training set and evaluated on the testing set for each gene set. Performance was assessed using accuracy, sensitivity, and specificity. Receiver operating characteristic (ROC) curves were plotted for top-performing gene sets, and area under curve (AUC) values were calculated using the *pROC* package to visualize predictive performance.

### Real-life patient cohort

Whole blood samples (2.5 ml) were taken from 36 active IBD patients and 30 healthy controls at the time of diagnosis using PAXgene Blood RNA System according to the manufacturer’s instruction. Participants had a diagnosis of IBD and healthy condition at the gastrointestinal and liver diseases clinic of Taleghani hospital, Tehran, Iran. All patients had undergone clinical and endoscopic evaluations at the time of sample collection. Disease activity was assessed using the endoscopic scores. Written informed consent was obtained from each participant and/or their legal guardian(s) and this research was approved by the ethical review board of Shahid Beheshti University of Medical Sciences, Tehran, Iran (IR.SBMU.RIGLD.REC.1402.028). Blood samples were also collected for standard clinical tests including C-reactive protein (CRP), white blood cells (WBC), vitamin D3, and erythrocyte sediment rate (ESR). Selected samples from patients with IBD including UC and CD were in the active phase of the disease.

### Total oxidant status assay

After centrifuging the whole blood for 10 min at 1000×g, the plasma was separated and stored at -80°C for preservation. Then, plasma samples were assayed at the earliest time points for levels of total oxidant status (TOS) by spectrophotometry of xylenol orange coupling with Fe3+ due to oxidation of Fe2+ according to the manufacturer’s instructions.

### Quantitative RT-PCR

Total RNA was extracted from whole blood samples according to the manufacturer’s instructions. RNAs were reverse transcribed into cDNA using AmpliQon SYBR Mix (Odense, Denmark). Then, using *GAPDH* as housekeeping control, qRT-PCR was performed using Qiagen Rotor-Gene 6000 real-time PCR cycler (Corbett, Hilden, Germany). The primers’ sequences manufactured by the Pishgam (Tehran, Iran) are shown in **Supplementary Table 1**. The Fold Change (FC) was calculated according to the 2^-ΔΔCT^ formula. IBD and control groups were compared with the Mann-Whitney u-test, and *p* < 0.05 was considered statistically significant. The data were expressed by the ± Standard Error of the Mean (SEM) and analyzed by the GraphPad Prism (version 8.4.3).

### Diagnostic panel evaluation in real-life cohort

To further assess the diagnostic potential of the final blood multi-mRNA panel, SVM was applied to Δct expression data using 6-fold cross-validation. The 66 samples were divided into six folds, each containing six IBD and five control participants. The model was trained on five folds and tested on the remaining fold. Performance was evaluated using accuracy, sensitivity, and specificity. ROC curves were plotted, and AUC values calculated to visualize the panel’s diagnostic power.

## Results

### Study design

In the discovery phase of this study, two microarray datasets, GSE94648 and GSE119600 (**Supplementary Tables 2**, **3**), were integrated to create a cohort of 132 patients with IBD (55 UC and 77 CD) and 60 healthy controls for the identification of DEGs in blood samples (**Supplementary Figure 1**). Using these DEGs, we performed functional analyses, including immune cell subpopulation analysis, GO enrichment, pathway enrichment, and network analysis.

To distinguish IBD-specific DEGs from those associated with general blood inflammation, a microarray dataset of rheumatoid arthritis samples (GSE93272; 65 RA patients and 43 controls, **Supplementary Tables 4**, **5**) was analyzed. DEGs common to both the RA vs. control and IBD vs. control comparisons were excluded to refine a list of IBD-specific candidate biomarkers. The LASSO algorithm was then applied to select gene biomarkers from the remaining DEGs capable of distinguishing IBD from non-IBD samples. A support vector machine model was used to evaluate the diagnostic performance of different biomarker combinations in the discovery cohort.

To further validate these findings, two independent in-silico test cohorts were analyzed: GSE169568 (**Supplementary Tables 6**, **7**), a microarray dataset comprising 110 IBD patients (58 UC and 52 CD) and 95 controls, and GSE166924 (**Supplementary Tables 8**), an RNA-sequencing dataset including 4 IBD patients (2 UC and 2 CD) and 11 controls.

For experimental validation, a real-life cohort consisting of 36 IBD patients (18 UC and 18 CD) and 30 healthy controls was recruited at Taleghani Hospital (**Table 1**). Independent two-tailed t-tests confirmed no significant differences in age (p = 0.8110) and sex (p = 0.8254) between IBD patients and controls, minimizing demographic bias. Clinical factors such as WBC count, hemoglobin (Hb) levels, vitamin D levels, CRP, and ESR were also compared. Although WBC, CRP, and ESR were higher in IBD patients, these differences were not statistically significant (p = 0.133, 0.355, and 0.549, respectively). However, Hb and vitamin D levels were significantly lower in IBD patients compared to controls (p = 0.00001 and p = 0.000003, respectively). Additionally, complete blood count (CBC) analysis revealed significantly higher neutrophil counts in IBD patients compared to controls (p < 0.001).

**Table 1:** Demographic and clinical characteristics of real-life patient cohort with inflammatory bowel disease and healthy condition.

Clinical Factors	Cnt (n = 30)	IBD (n = 36)
Sex (Male/Female)	15 Male/15 Female	19 Male/17 Female
Disease Duration, Median years (IQR)	–	5.5 (0.7 – 16)
Age, Median years (IQR)	35 (18 – 58)	32.5 (18 – 62)
Erythrocyte Sediment Rate, mm/hr, (IQR)	15 (5 – 30)	30 (13 – 65)
C-Reactive Protein, mg/l, (IQR)	12 (8 – 47.2)	17.5 (11 – 96)
BMI	26.62 (20.12–30.45)	28.89 (25–37.71)
Hb, g/dl, (IQR)	14.2 (13.43 – 15.4)	12.1 (8.95 – 13.1)
WBC, ×1000/mm^3^, (IQR)	7.3 (6.20 – 9.20)	8.97 (6.63 – 12.6)
Vitamin D, ng/ml, (IQR)	50.5 (37 – 61)	29.5 (14.5 – 54)
Neutrophils, 10^6^/μL, (IQR)	4.23 (3.4 – 5.2)	6.01 (4.8 – 7.9)

IBD, Inflammatory bowel diseases patients.

Cnt, Healthy control.

**Figure 1:**
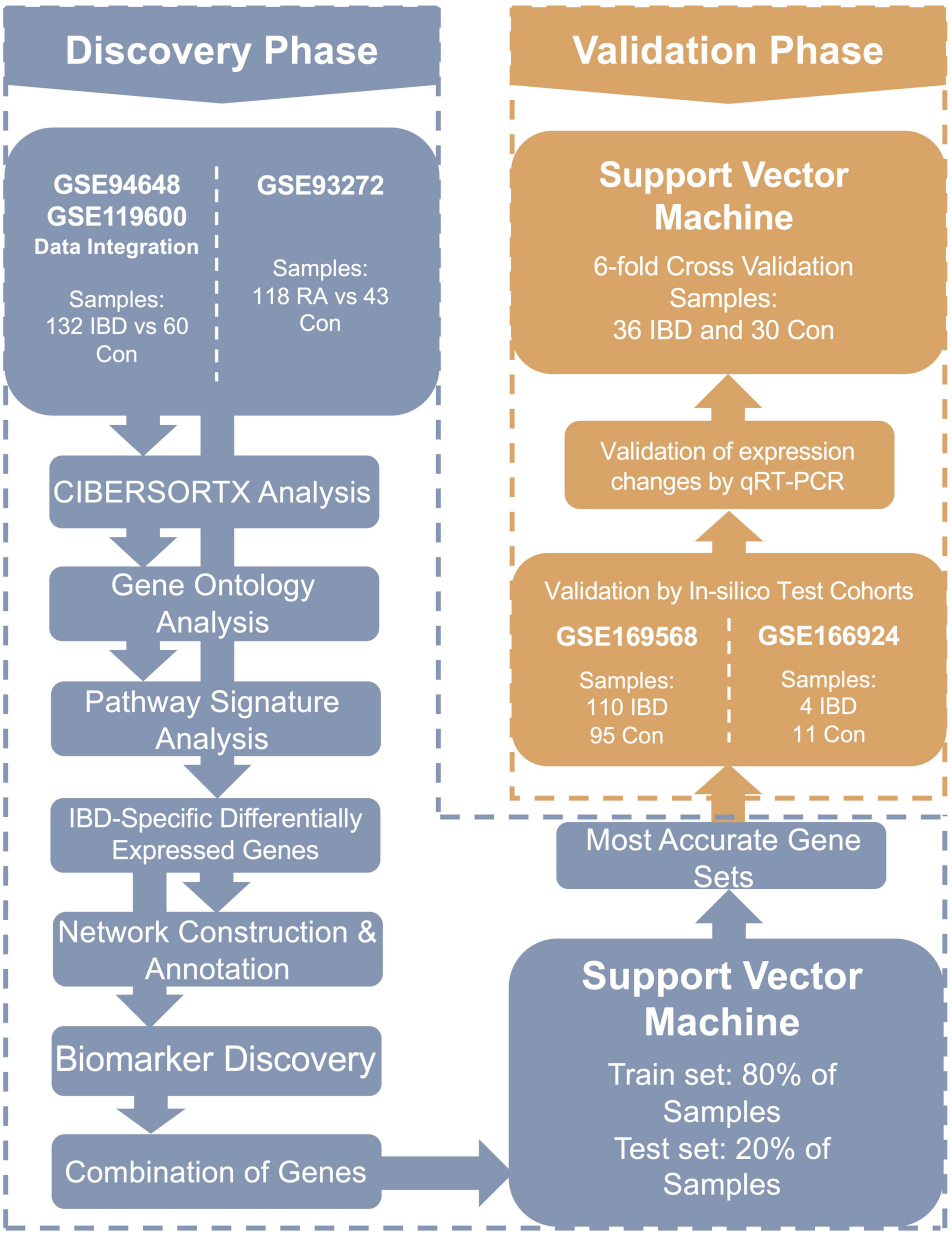
The flowchart of the current research. In this study, transcriptomes of whole blood samples from 254 participants were analyzed to identify diagnostic and pathogenic biomarkers as well as involved signaling pathways in IBD. IBD, Inflammatory bowel diseases patients.

To evaluate the diagnostic performance of the selected biomarkers experimentally, gene expression levels were measured by qRT-PCR in whole blood samples from the 66 participants. An SVM model was again applied to the qRT-PCR data to assess the diagnostic panel’s ability to distinguish IBD from controls. The overall study workflow is summarized in **Figure 1**.

### IBD-specific blood immune cell subpopulations

**Figure 2:**
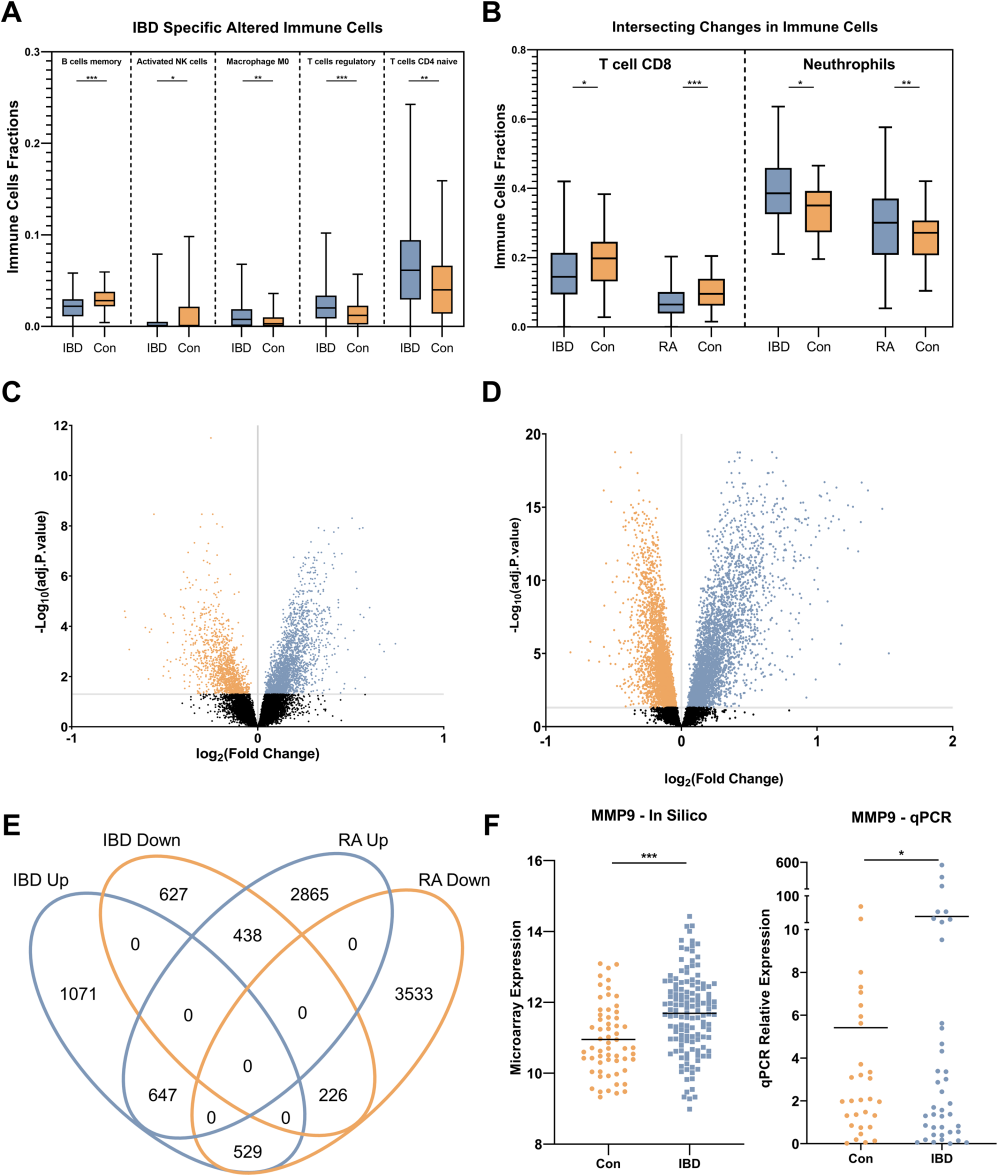
Immune cell profile and gene expression analysis. **(A)** alterations of IBD-specific immune cell subpopulation include increased proportions of M0 macrophages, T regulatory, and T CD4 naïve cells, and decreased fractions of memory B lymphocytes and activated natural killer (NK) cells. **(B)** Common immune cell profile changes in IBD and RA compared to control individuals including, reduction of T CD8 cells and neutrophil subpopulations. **(C)** Volcano plots show the number of statistically (Adjusted p. < 0.05) significant differentially expressed genes in IBD. **(D)** Volcano plots show the number of statistically (Adjusted p .<0.05) significant differentially expressed genes in RA. **(E)** The Venn diagram shows the intersecting and specific statistically significant differentially expressed genes in IBD and RA when compared to the control. **(F)** The expression alteration of MMP9 as the most differentially expressed gene in both in silico and the real-life cohort. Con, Healthy control; IBD, Inflammatory bowel diseases patients; RA, rheumatoid arthritis patients. * adjusted p.value < 0.05; ** adjusted p.value < 0.01; *** adjusted p.value < 0.001.

CIBERSORTx was used to deconvolute gene expression microarray data to determine the distinct immune cell subpopulation changes in IBD. Specifically, M0 macrophages, T regulatory (Treg), and CD4 naïve T cells had significantly increased populations in IBD while memory B lymphocytes and activated natural killer (NK) cells were downregulated compared to control individuals (**Figure 2A**). Furthermore, CD8 T cell and neutrophil subpopulations were increased in IBD patients compared to healthy controls, similar to what was observed in RA patients (**Figure 2B**). Notably, the CIBERSORTx-inferred increase in neutrophil populations in IBD patients was consistent with neutrophil counts obtained from CBC analysis of the real-life patient cohort. Treg and CD4 naïve T cells were also detected to alter significantly in RA patients compared to controls while having a converse pattern in which these cells had reduced fractions in RA (data not shown).

### Identification of statistically significant altered genes

Using the discovery microarray cohorts (GSE94648 and GSE119600) for IBD, and GSE93272 for RA, blood transcriptomes of patients were compared to healthy individuals, and DEGs were identified by using FDR < 0.05. In IBD, 3538 significant DEGs were found, including 2247 upregulated and 1291 downregulated (**Figure 2C**). In RA, 8238 significant DEGs were found, including 3950 overexpressed and 4288 downregulated (**Figure 2D**). Intersecting DEGs from two conditions showed 1840 overlapping genes (**Figure 2E**), and 1698 IBD-specific genes, among which *MMP9* expression had the highest change. This was verified in the real-life cohort by qRT-PCR (**Figure 2F**).

### Cellular respiration, the most altered pathway

**Figure 3:**
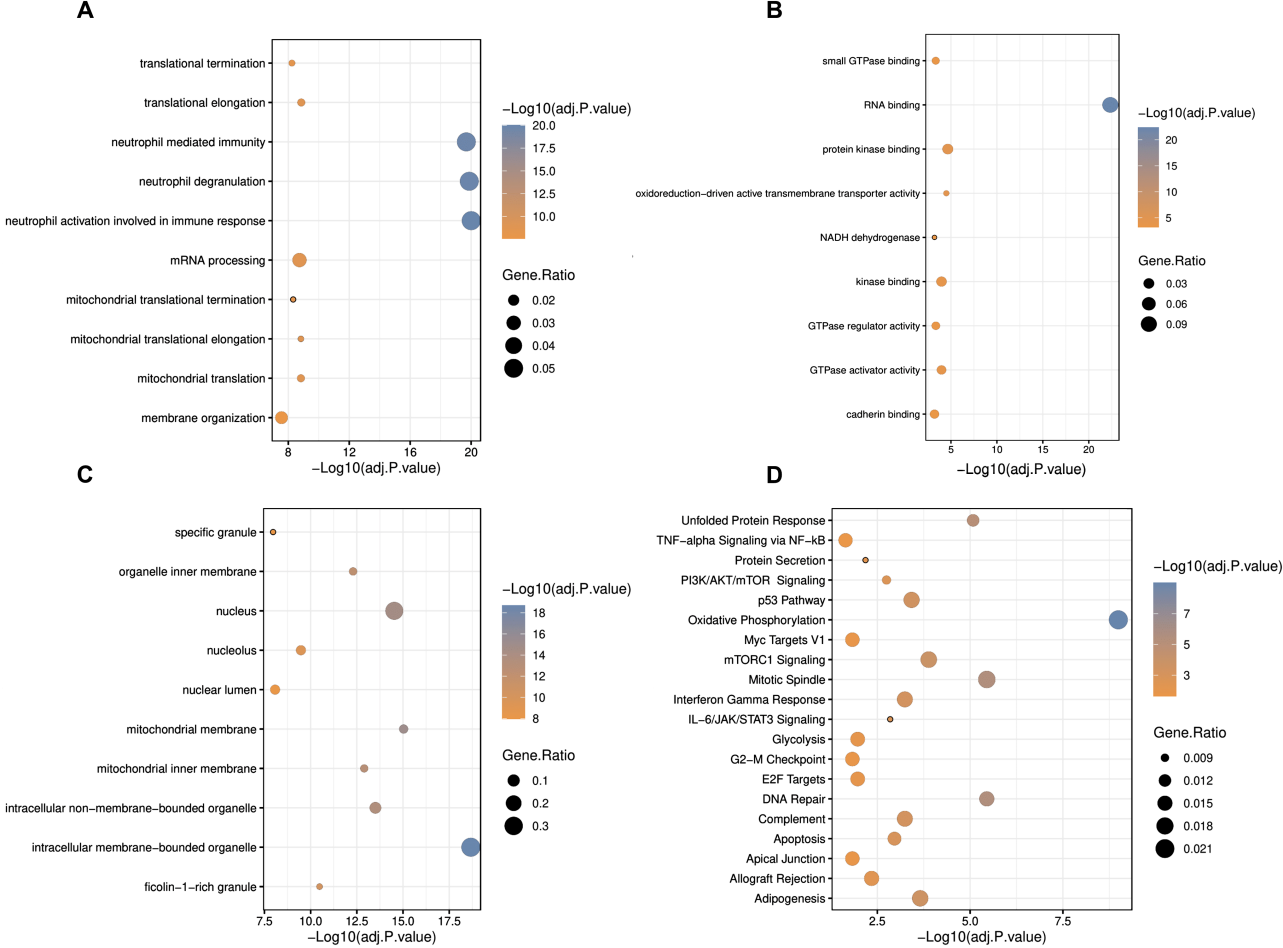
Gene ontology and pathway enrichment analyses of differentially expressed genes in IBD. **(A)** Biological process, **(B)** molecular function, **(C)** cellular component, and **(D)** pathway analysis in GOR and MSigDB. Statistically significant GOs and pathways were detected by considering adjusted p. <0.05 as the criterion. * adjusted p.value < 0.05; ** adjusted p.value < 0.01; *** adjusted p.value < 0.001.

Functional enrichment analysis was carried out to understand the disease mechanisms associated with DEGs in IBD. GO analysis indicated neutrophil activation involved in immune response, neutrophil degranulation, and neutrophil-mediated immunity for BP (**Figure 3A**), RNA binding, protein kinase binding, and oxidoreduction-driven active transmembrane transporter activity for MF (**Figure 3B**), and intracellular membrane-bounded organelle, mitochondrial membrane, and nucleus for CC (**Figure 3C**), as hallmarks, respectively. Besides, the DEGs were shown to be enriched in mitochondrial-associated cellular processes and components, suggesting an important role for this organelle in blood cells of IBD patients. Pathway analysis identified oxidative phosphorylation as a critical pathway associated with blood inflammation (**Figure 3D**), suggesting dysregulation of cellular respiration processes in the blood cells of IBD patients.

### Oxidative phosphorylation disruption in the blood of IBD patients

**Figure 4:**
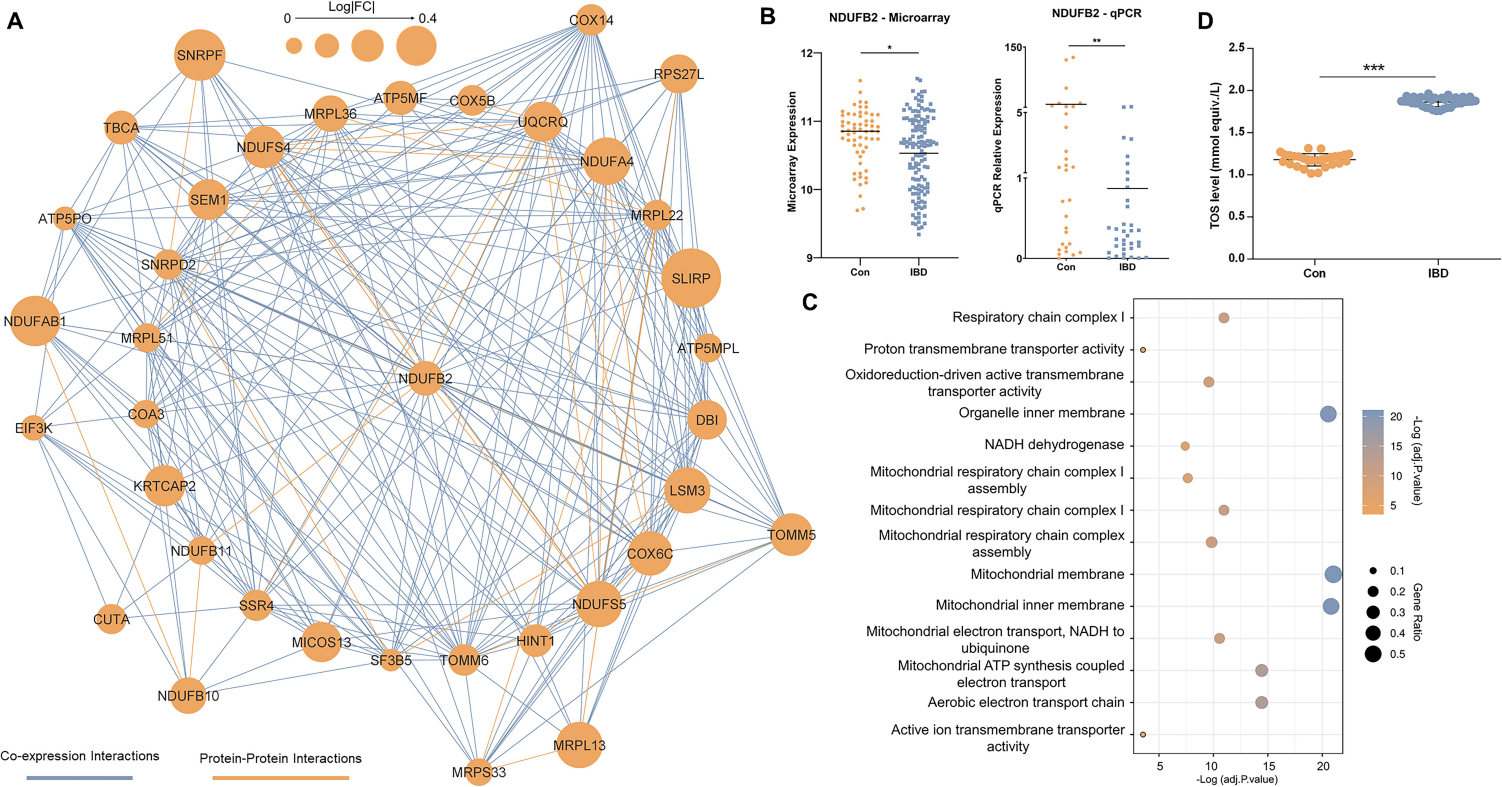
Network analysis. **(A)** The major model includes 37 genes communicated through 302 edges. Coexpression and protein-protein interactions are shown in light blue and orange colors, respectively. The size of nodes is determined by the value of expression alteration for the relevant gene. **(B)** Expression changes in NDUFB2 as the hub node in the major model. **(C)** Gene ontology analysis of genes in the major network model. Statistically significant GOs were detected by considering adjusted p. < 0.05 as the criterion. The components of the figure are made by R and Cytoscape. **(D)** Total oxidant status (TOS) levels measured in the blood of control individuals (Con) and IBD patients (IBD). TOS levels were significantly elevated in IBD patients compared to controls (* adjusted p.value < 0.05; ** adjusted p.value < 0.01; ***p<0.001). Data are shown as individual data points with mean ± SEM.

A network of co-expressed genes, which also includes protein interactions, was constructed in Cytoscape, comprising 560 nodes interacting through 1828 edges. Using the *CytoHubba* plugin, the top 50 nodes according to the degree were discovered including, *UQCRQ*, *MRPL51*, *NDUFA4*, *LSM3*, and *NDUFB2* as the most interactive genes (**Supplementary Table 9**). Among these 50 nodes, a cohesive module was found with 302 edges between 37 nodes, which all were interestingly downregulated (**Figure 4A**). Analyzing the model with the *CentiScape2.2* plugin revealed *NDUFB2* as the hub node based on radiality, showing the node as the regulatory of the entire network (**Supplementary Table 10**).

Microarray and qRT-PCR expression data indicated an association between downregulation of *NDUFB2* in blood and IBD pathogenesis (**Figure 4B**). Analyzing this module in Pathview showed oxidative phosphorylation as the most significant associated pathway (**Supplementary Figure 2**). Similarly, GO analysis showed enrichment for aerobic electron transport chain (BP), oxidoreduction-driven active transmembrane transporter activity (MF), and mitochondrial membrane localization (CC) (**Figure 4C**).

These findings suggest that mitochondrial respiratory function is disrupted in the blood cells of IBD patients, with NDUFB2 playing a key role as part of the NADH dehydrogenase complex (Complex I) in the electron transport chain. Specifically, the downregulation of NDUFB2 may impair electron transfer during oxidative phosphorylation, leading to increased electron leakage and elevated production of reactive oxygen species (ROS), thereby contributing to oxidative stress and systemic inflammation observed in IBD.

To test these findings, we measured TOS levels in blood samples from our real-life patient cohort. Consistent with the hypothesis of elevated oxidative stress, TOS levels were significantly higher in IBD patients compared to healthy controls (p < 0.001) (**Figure 4D**), indicating of increased oxidative stress in the blood samples of IBD patients.

### Identification of a multi-mRNA IBD diagnostic biomarker panel

Among IBD-specific DEGs, 25 (14 upregulated and 11 downregulated) had |log_2_FC| > 0.5, among which *CD160* and *MMP9* had the most changes. Applying LASSO to these 25 genes identified 15 candidate diagnostic biomarkers, amongst others *EIF5*, *IL4R*, and *SLC9A8* had the greatest discriminative potentiality according to the LASSO absolute value (**Supplementary Table 11**). By using the *COM* function in R, 148,395 gene sets (three to eight genes) were designed from these 15 diagnostic biomarkers. The SVM monitored the accuracy of each gene set by training on 80% of the samples and testing on the remaining 20%. For each gene set category (based on the number of genes), the most accurate panel was identified (**Table 2**).

**Table 2:** The most accurate gene sets were revealed by support vector machine using 80% of patients in the train set and 20% in the test set.

Gene in Panel	Accuracy	Sensitivity	Specificity
EIF5A, IL4R, SLC9A8	0.84	0.856	0.8
EIF5A, SLC9A8, CD160, PDZK1IP1	0.86	0.879	0.817
EIF5A, IL4R, SLC9A8, CD160, PDZK1IP1	0.865	0.894	0.8
EIF5A, IL4R, SLC9A8, SLC26A8, CD160, PDZK1IP1	0.865	0.886	0.817
EIF5A, IL4R, SLC9A8, SLC26A8, MMP25, CD160, PDZK1IP1	0.87	0.902	0.8
EIF5A, IL4R, SLC9A8, GZMB, CTSW, FGFBP2, CD160, MMP9	0.865	0.894	0.79

**Figure 5:**
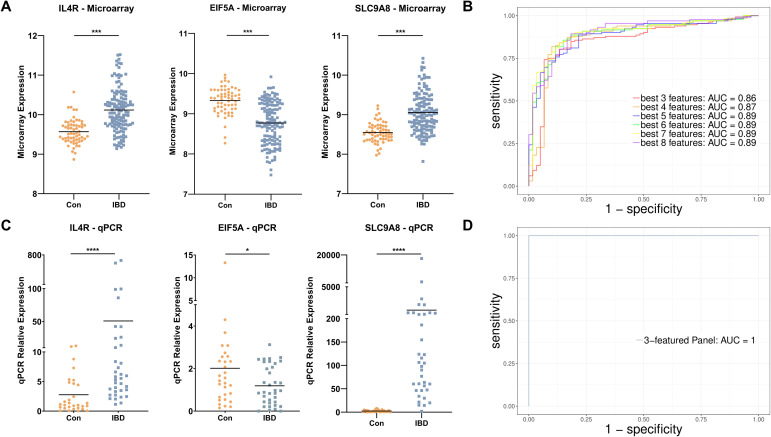
Diagnostic gene set discovery and verification. **(A)** The expression level of *IL4R*, *EIF5A*, and *SLC9A8* in IBD patients and control individuals in microarray data. **(B)** ROC curve construction and AUC calculation for the most accurate gene sets revealed an AUC of 0.86 for the three-biomarker panel in the discovery cohort. **(C)** The expression level of *IL4R*, *EIF5A*, and *SLC9A8* in IBD and healthy controls based on qRT-PCR data of the real-life patient cohort. **(D)** ROC curve construction and AUC calculation for the three-mRNA gene set revealed an AUC of 1 in real-life cohort data. Con, Healthy control; IBD, Inflammatory bowel diseases patients. * adjusted p.value < 0.05; *** adjusted p.value < 0.001; **** adjusted p.value < 0.0001.

Although four- to eight-mRNA sets were more accurate, the difference between their accuracy and the accuracy of the three-mRNA panel was not considerable; hence, the three-mRNA biomarker panel which is more economical for patients was revealed to be the best diagnostic panel for IBD with the accuracy of 84% (sensitivity 85.6% and specificity 80%). The three-biomarker panel contains *EIF5A*, *IL4R*, and *SLC9A8* that interestingly had the higher LASSO values compared to other features and were altered with fold changes of 0.68, 1.42, and 1.46, respectively in IBD (**Figure 5A**). The area under ROC curves were 0.86 and 0.87 for three- and four-RNA biomarker panels, respectively and 0.89 for five- to eight-gene sets (**Figure 5B**).

To further validate the differential expression of the biomarkers in independent external datasets, we analyzed two additional publicly available blood-based cohorts: GSE168568 (microarray data) and GSE166924 (RNA-sequencing data). In GSE168568 (n=205), IL4R and SLC9A8 showed significant upregulation in IBD patients compared to controls, while EIF5A did not reach statistical significance (FDR < 0.1), likely due to higher variability. In GSE166924 (n=15), EIF5A and IL4R were significantly differentially expressed in IBD patients with a consistent expression trend. While SLC9A8 also showed consistent expression trends it did not reach statistical significance, possibly due to the limited sample size (**Supplementary Figure 3**).

To further assess the expression and discriminative potentiality of the three selected diagnostic biomarkers, qRT-PCR was applied to whole blood samples of patients in Taleghani Hospital. At first, the statistically significant higher expression of *IL4R* and *SCL9A8* and lower expression of *EIF5A* were verified in IBD patients (**Figure 5C**). To assess the diagnostic performance of the three-mRNA, the SVM classifier was used through a 6-folds cross-validation approach and showed an impressive accuracy of 99% (sensitivity 100% and specificity 97%) and AUC of 1 (**Figure 5D**). To further evaluate the differentiative performance of IL4R, EIF5A, and SCL9A8, each was used as a principal component. PCA plots demonstrated their strong ability to distinguish IBD patients from controls (**Supplementary Figure 4**).

## Discussion

Despite advances in using clinical symptoms and serological or stool markers for IBD diagnosis, these methods lack sensitivity, with colonoscopy remaining the gold standard despite its invasiveness and side effects. Here, we used blood transcriptomics to identify biomarkers for non-invasive detection of IBD. We began by integrating microarray gene expression profiles from multiple cohorts from different regions. This approach not only reduced the false positive rate and one-sided results but also increased the sample size to enhance variability and power of computation. Then, using LASSO and SVM, which are well-suited for analyzing high-dimensional data sets with limited sample sizes due to utilizing regularization strategies to pinpoint the most pertinent features (25, 26), a three-mRNA panel of *IL-4R*, *SLC9A8*, and *EIF5A* was developed as the most clinically applicable gene set with an average accuracy of 91.5% and AUC of 0.93 in cohorts. Numerous studies have analyzed the transcriptomes of intestinal biopsies obtained from IBD patients to identify disease biomarkers and explore the underlying intestinal inflammation and treatment responses (27–30), which is an invasive strategy like colonoscopy that is routinely used as a gold standard method for IBD diagnosis. However, this study provides an accurate non-invasive tool for IBD diagnosis by whole blood transcriptome analysis.

Among the predictors, the IL-4 receptor (IL-4R) plays a central role in immune regulation by mediating the effects of IL-4, a key cytokine involved in the differentiation of naïve CD4+ T cells into Th2 cells and the alternative activation of macrophages. These processes are crucial for balancing pro-inflammatory and anti-inflammatory responses in the immune system. In the context of IBD, dysregulation of IL-4R expression and signaling pathways may contribute to immune imbalances, driving chronic inflammation (31–33). Considering the possible role of IL-4R in the inflammatory pathways, the measurement of IL-4R in the serum is reasonable to be diagnostically important.

The second marker; SLC9A8, known as NHEs, constitutes one of the four Na+/H+ exchangers with the predominant expression in the trans-Golgi, which plays a crucial role in protein trafficking and the morphology of endosomes (34). Although SLC9A8 is localized on the apical membrane of intestinal and renal epithelial cells (35, 36), we found for the first time that it is also enriched in the blood of IBD patients and could serve as potential diagnostic biomarker. However, further research is needed to fully understand the role of SLC9A8 in IBD and how it may be targeted for the treatment.

Eukaryotic translation initiation factor 5A, or EIF5A, is another biomarker identified for its role in the translation elongation and termination of specific protein subsets (37). It has been demonstrated that overexpressed EIF5A initiates apoptosis in colorectal cancer (CRC) cells, such as HTC116 and HT29 while EIF5A knockdown leads to the decreased expression of tumor suppressor p53 (38). Furthermore, down regulation of Klf5 followed by depletion of EIF5A results in excessive accumulation of ROS, mitochondrial dysfunction, and senescence. in vascular disorders (39). These findings are consistent with the elevated TOS (oxidative stress) and the downregulation of NDUFB2 observed in IBD patients in our study. *Tan et al.* showed that EIF5A in CD8^+^ T cells significantly contributes to the cell survival and the production of proteins involved in cell proliferation and cytotoxicity (40). Although dysregulation of EIF5A has been associated with cancer development (38) as well as inflammation diseases (41, 42), the role of EIF5A in IBD remains to be elucidated (37).

Among the differentially expressed genes, matrix metalloproteinase-9 (MMP9) exhibited the highest fold change (1.67) in IBD patients compared to controls. MMPs are globally linked to extracellular matrix degradation, contributing to ulceration and tissue remodeling (43). Activated circulating neutrophils are major sources of peripheral blood MMPs (44, 45). Also, elevated neutrophil counts, often detected in routine blood tests of IBD patients have been shown to correlate with disease activity (46). This is consistent with the higher neutrophil subpopulations observed by CBC and CIBERSORTx in the blood of patients with IBD in our study. Neutrophils play a key role in the acute inflammatory response in IBD by releasing ROS, proteolytic enzymes, and proinflammatory cytokines that can exacerbate tissue damage. Increased neutrophil infiltration into the intestinal mucosa has also been associated with histologic severity and worse clinical outcomes (47, 48). Elevated peripheral neutrophil counts reflect ongoing systemic inflammation and are increasingly recognized as a potential non-invasive biomarker for disease flare prediction and monitoring (49). Together, these findings suggest that the mRNA expression level of MMP9 in blood is linked to neutrophil activation and may serve as a surrogate marker of disease severity in autoimmune conditions such as IBD.

Deconvolution of expression data using CIBERSORTx revealed alterations in both innate and adaptive immune cell subpopulations, supporting recent insights that innate immune responses may be as critical, if not more so, than adaptive immune responses in triggering gut inflammation in those with IBD (50). Particularly in IBD, the population of M0 macrophages, Tregs, and T CD4^+^ naïve cells increased, while the number of memory B cells and activated NK cells decreased. Although these findings require experimental validation, some of them are supported by existing literature on immune cell alterations in IBD. Currently, the increase in CD4^+^ T cells exhibiting an activated effector memory phenotype is evident in the peripheral blood of adult and pediatric patients affected by IBD (51–53). It has been reported that CD4^+^ naïve T cells and the memory/effector CD4^+^ T-cell population were significantly activated within inflammation conditions and secreted various proinflammatory cytokines such as IL-1β, IL-4, IL-6, IL-21, and TGF-β (54).

Network, GO, and pathway analyses showed oxidative phosphorylation in mitochondria with *NDUFB2* and *NDUFA4* as the main regulators. NDUFB2 is a gene that codes for a subunit of mitochondrial complex I, which is responsible for electron transport from NADH to the respiratory chain and has a critical role in mitochondrial function (55). Downregulation of *NDUFB2* and other mitochondrial genes, such as NDUFA4, UQCRQ, and MRPL5, suggests a possible mechanistic link between mitochondrial dysfunction and the pathogenesis of IBD. It is known that the respiratory chain complex I dysfunction could be associated with the production of ROS (56). These findings are consistent with our observations of increased neutrophil populations and elevated TOS in IBD patients. Neutrophils are major producers of ROS during inflammation (57), and mitochondrial dysfunction—particularly impairment of complex I—can further enhance ROS production, leading to oxidative stress (58). The downregulation of mitochondrial complex I components such as NDUFB2 and NDUFA4 suggests compromised mitochondrial respiration, which could contribute to excessive ROS accumulation and inflammatory responses in the blood.

Mitochondrial dysfunction has been increasingly recognized as a key contributor to systemic inflammation. Several studies have shown that impaired mitochondrial respiration and excessive ROS generation in peripheral blood cells are associated with inflammatory conditions such as sepsis and rheumatoid arthritis (59, 60). In IBD specifically, Dashdorj et al. showed that mitochondrial dysfunction followed by ROS production can be an initiator of inflammation and introduced MitoQ, a mitochondria-targeted antioxidant as a therapeutic agent ameliorating inflammation in a colitis mouse model (61). Likewise, using conplastic mice with nuclear but different mitochondrial genomes, it has been shown that increased activity of OXPHOS and higher production of ATP could suppress colitis in mice, suggesting it as a therapeutic approach for IBD (62). This abnormalities extend beyond the intestinal mucosa and are detectable in circulating immune cells (63). Reduced mitochondrial membrane potential, elevated oxidative stress, and impaired energy metabolism have been reported in peripheral blood mononuclear cells (PBMCs) of IBD patients (64). Dysfunctional mitochondria in blood immune cells may therefore not only reflect disease activity but also actively contribute to systemic inflammation by promoting ROS overproduction and inflammatory cytokine release. These findings support the hypothesis that mitochondrial dysfunction in blood cells may represent an important driver of blood inflammation and oxidative stress in IBD.

Moreover, the disruption of oxidative phosphorylation pathway observed in our data aligns with a state of heightened cellular stress and energy demand during active inflammation. Although further experimental studies are needed, these findings support a model in which mitochondrial dysfunction, neutrophil activation, and oxidative stress are interconnected processes contributing to the pathogenesis of IBD.

In summary, we developed a robust three-mRNA biomarker panel comprising IL-4R, SLC9A8, and EIF5A for the non-invasive diagnosis of IBD. While this panel demonstrates high accuracy, its performance for clinical application needs to be validated in larger, more diverse cohorts. Generating multi-center RNA-seq data from larger cohorts followed by application of machine learning algorithms will enable us to identify subtype-specific biomarkers differentiating UC from CD. Additionally, our findings highlight CD4+ T cells as a key immune cell population exhibiting IBD-specific alterations, and neutrophils as a broader marker of systemic inflammatory responses in the blood. Our analysis also indicated that the downregulation of NDUFB2 occurs in blood samples of IBD patients, linking this alteration to key mechanisms such as mitochondrial dysfunction, impaired oxidative phosphorylation, and excessive ROS production, ultimately contributing to blood inflammation. These findings suggest new avenues for therapeutic exploration, with mitochondrial pathways representing promising targets for future treatment development in IBD and related inflammatory conditions. We also identified MMP-9 as a potential marker for disease activity, though its lack of specificity, as it is also elevated in RA, limits its diagnostic application for IBD alone.

## Data Availability

The datasets presented in this study can be found in online repositories. The names of the repository/repositories and accession number(s) can be found in the article/**Supplementary Material**.
