# Supplementary material for: Biomarker discovery for non-invasive diagnosis of inflammatory bowel disease using blood transcriptomics
Source: Front Immunol. 2025 Jun 20;16:1570374. doi: 10.3389/fimmu.2025.1570374 (PMC12226589; doi:10.3389/fimmu.2025.1570374)
Supplement: Supplementary file 1 [file DataSheet1.docx]

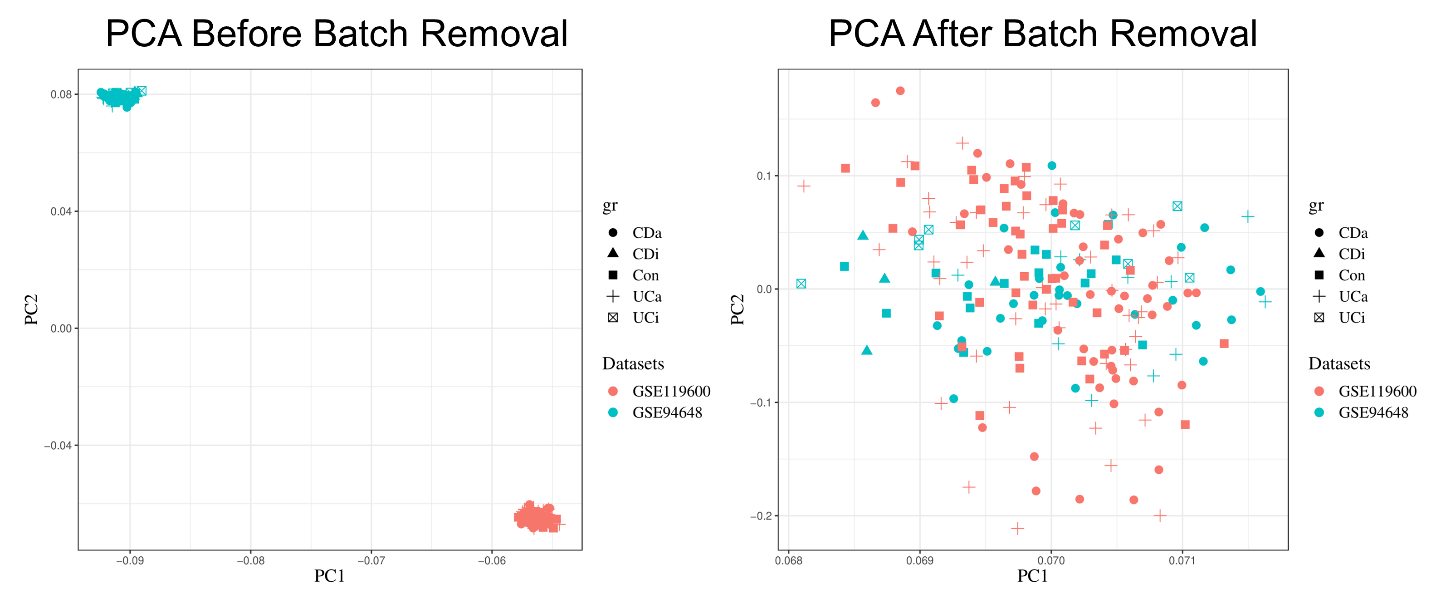


**Supplementary Figure 1.** Batch effect removal was performed to integrate IBD data and generate a metadata by using *ComBat* function in R.


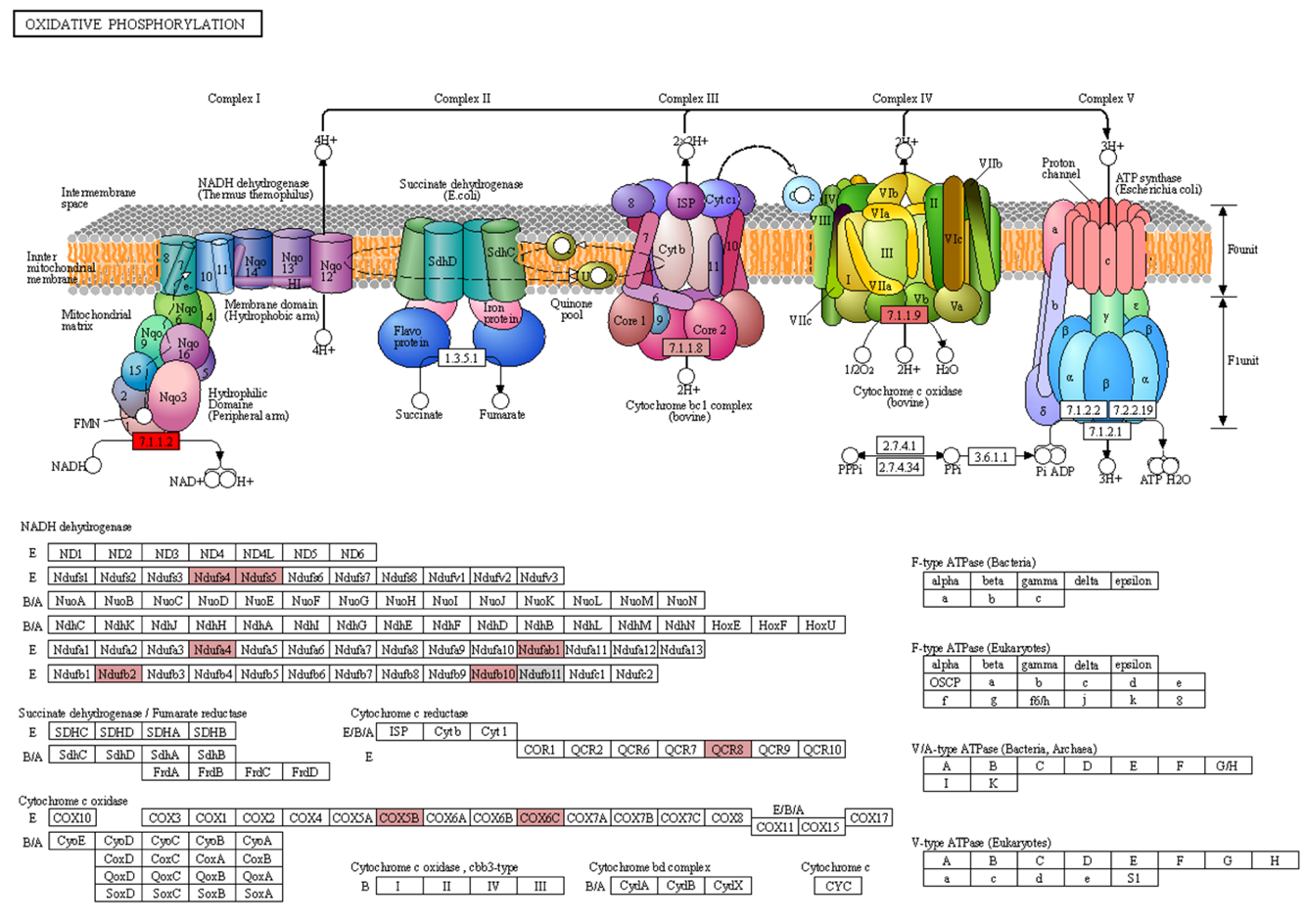


**Supplementary Figure 2.** Network analysis in pathview revealed oxidative phosphorylation as the signature pathway involved in IBD pathogenesis in whole blood. Highlighted genes in and below the graph are those that were found in the major network (The figure is created using <https://pathview.uncc.edu/>(1)).


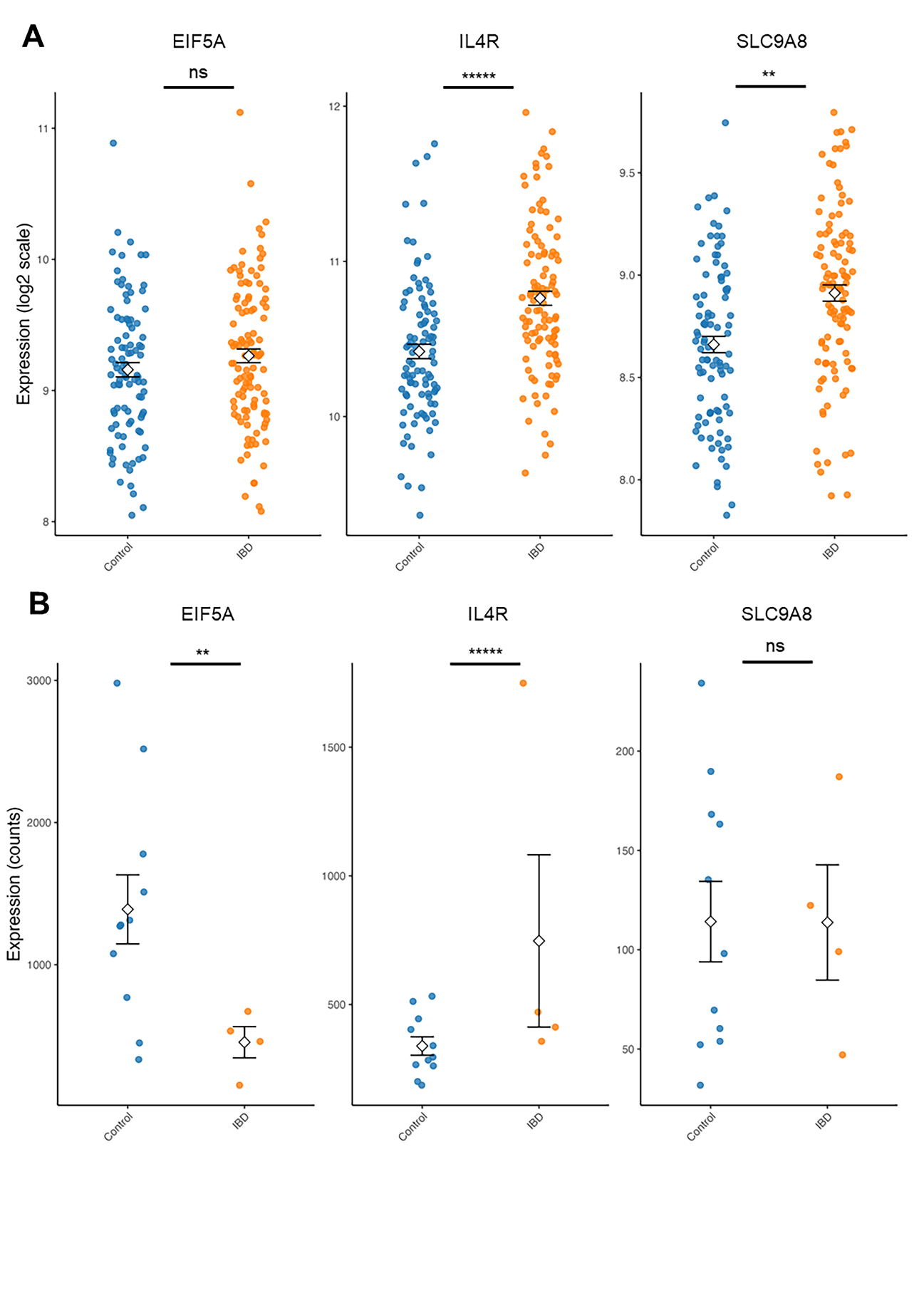


**Supplementary Figure 3.** Validation of biomarker expression in independent test cohorts. (A) Expression levels of selected biomarkers (EIF5A, IL4R, and SLC9A8) in the GSE169568 microarray dataset comparing IBD patients and control individuals. (B) Expression levels of the same biomarkers in the GSE166924 RNA sequencing cohort. Statistical significance was determined using appropriate tests, with p-values indicated as follows: ns, not significant; **, p < 0.01; *****, p < 0.00001.


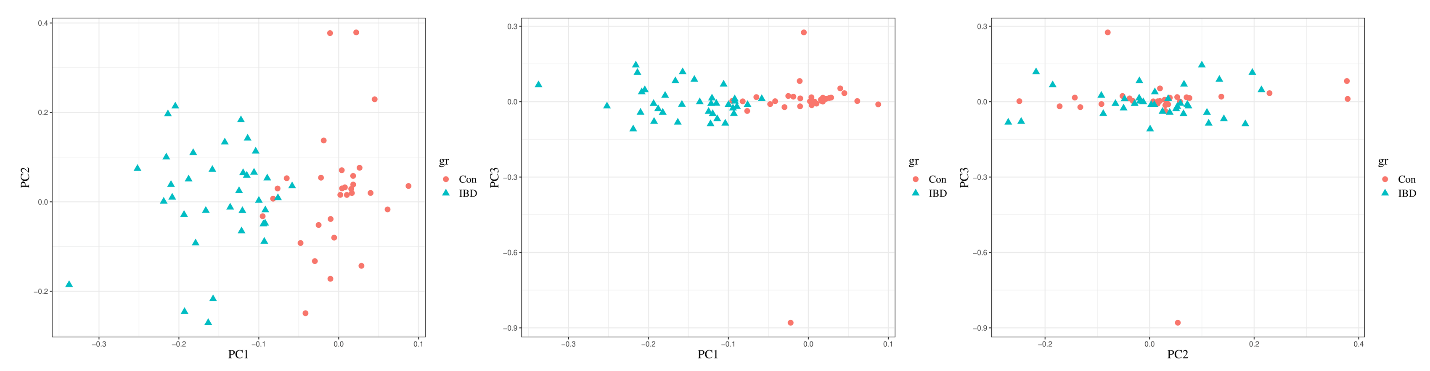


**Supplementary Figure 4.** Principal component analysis of real-life patients based on the expression of *IL4R*, *EIF5A*, and *SLC9A8* indicated a noticeable discriminative performance of these genes.

**Reference:**

1. Luo W, Friedman MS, Shedden K, Hankenson KD, Woolf PJ. GAGE: generally applicable gene set enrichment for pathway analysis. BMC bioinformatics. 2009;10:1-17.
